# Effects of combination of strength and balance training on postural control and functionality in people with chronic ankle instability: a systematic review and meta analysis

**DOI:** 10.1186/s13102-024-00845-1

**Published:** 2024-04-09

**Authors:** Yuying Su, Wei Li, Changbo Pan, Yu Shi

**Affiliations:** 1 School of Strength and Conditioning Training, Beijing Sport University, Beijing, China; 2 Physical Education College, Bohai University, Jinzhou, Liaoning, China

**Keywords:** Balance training, Strength training, Functionality, Chronic ankle instability, Dynamic balance

## Abstract

**Aim:**

To identify the effects of strength and balance training on dynamic balance and patient reported outcomes in people with chronic ankle instability(CAI).

**Method:**

Five databases(CNKI, WanFang, Web of Science, EBSCO-SPORTD and PubMed were searched in September 2022. The search was conducted on randomized controlled trials(RCTs) that the effects of strength training, balance training and combination of strength and balance training in people with chronic ankle instability compared to a control group. Using Review Manager 5.3 and Stata-SE 15 to conduct Meta-analysis on the included literature. methodological quality and risk of bias were assessed by using the PEDro scale.

**Results:**

A total of 33 Chinese and English RCTs document were screened and 1154 patients with CAI were included in the study. Compared with control group, strength training, balance training and combination of strength and balance training demonstrated to be more effective in terms of improving patient reported outcomes(strength training: SMD = 0.80, 95%CI = 0.39–1.22; balance training: SMD = 0.79, 95%CI = 0.41–1.17; combination of strength and balance training: SMD = 1.28, 95%CI = 0.57, 1.99). Subgroup analysis: Intervention for 6 weeks, more than 3 times a week and more than 30 min each time were the best rehabilitation programs to improve CAI patientreported outcomes. Compared with control group, balance training demonstrated to be more effective in terms of improving Star Excursion Balance Test (SEBT)((anterior: SMD = 0.71, 95%CI = 0.03–1.40; posterolateral: SMD = 0.84, 95%CI = 0.22–1.46; posteromedial: SMD = 0.88, 95%CI = 0.45–1.32). However, strength training and combination of strength and balance training had no improvement effects on SEBT.

**Conclusions:**

Available evidence showed that, results of the comparison between balance training versus strength training suggest that the combination of strength and balance training achieves greater benefits for patient reported outcomes and balance training could bring greater benefits to dynamic balance. Strength training should be used cautiously in clinic to improve the dynamic balance in individuals with CAI.

**Trial registration:**

(http://www.crd.york.ac.uk/PROSPERO, Registration No. CRD42022371396).

## Introduction

Ankle sprains are one of the most common types of injuries in daily life, often occurring in acute sports events such as basketball and soccer, as well as in daily rough roads. Among the sports injuries with data statistics, 25% of sports injuries were caused by ankle sprain in different degrees [1]. Minor ankle sprains can be effectively treated with non-surgical conservative treatment [2]. However, because many patients do not pay enough attention to ankle sprain in the rehabilitation stage or lack of necessary exercise rehabilitation means, as many as 70% of patients has been habitual sprain and instability in the later stage, and 40%-50% of patients would develop chronic ankle instability (CAI) [2, 3]. CAI refers to the structural or functional deficiencies in the ankle joint and surrounding tissues, resulting in ankle joint instability and limited joint movement, with recurrent sprains as the main characteristic [4]. The main clinical symptoms of CAI include muscle weakness, persistent pain, loss of control, ligament laxity, functional decline, and repeated sprains, accompanied by cartilage damage and synovitis, severely affecting the quality of daily life [5].

Generally, CAI could be rehabilitated by conservative methods such as ankle fixation, physical therapy and exercise therapy. Exercise therapy is an important treatment modality for the rehabilitation of CAI in the later stage [6]. Studies had shown that dynamic balance, proprioception, fibular reaction time and lack of valgus strength were the main cause of CAI symptoms [7]. Strength and balance training were the most commonly used in rehabilitation training. The aim of strength and balance training rehabilitation is to correct modifiable deficits such as reduced muscle strength, decreased neuromuscular control, impaired proprioception, altered gait pattern, and restricted range of motion that are observed in individuals with CAI [8, 9].

Previous research has suggested some benefits from strength and balance training for improving dynamic balance and patient reported outcomes in individuals with CAI [10–12]. But, the comparison of effectiveness between strength training, balance training and combination of strength and balance training on dynamic balance and self-reported function for CAI are still unclear [8]. We need to evaluate the impact of three types of exercise interventions on dynamic balance and self-reported function. We also need to consider which exercise mode, intervention load, intervention time, and intervention cycle can better treat chronic ankle instability symptoms. These factors are important for clinical development of exercise rehabilitation programs [13]. In view of the above, the aim of this study was to systematically review the available evidence on the effects of the various therapeutic physical exercise interventions (i.e. strength training, balance training and combination of strength and balance training) in individuals with CAI. Furthermore, meta-analysis was conducted to determine the most effective treatment for improving patient reported outcomes and dynamic balance in this population.

## Methods

### Data sources and search strategies

The literature search for this study was conducted independently and blindly by two researchers, strictly following the PRISMA statement for meta-analyses. The search databases included Wanfang, CNKI, Web of Science, PubMed, and EBSCO-SPORTD. The last search was conducted on September 23, 2022. The literature data is retrieved in both Chinese and English. A secondary search of the reference lists of selected articles was conducted to prevent the omission of important literature. This study was registered in the Research Registry (http://www.crd.york.ac.uk/PROSPERO, Registration No. CRD42022371396). Derived from the explosion search, the final search strategy was(“chronic ankle instability” OR “ankle instability” OR “function ankle instability”) AND (“exercise intervention” OR “strength training” OR “balance training” OR “neuromuscular control training”).

### Selection criteria

The studies were included in this review if they met the following criteria:

- (1) Participants: individuals with CAI, According to the standards of the International Ankle Federation, patients who meet the following conditions are included as CAI patients: 1) history of at least 1 ankle sprain(excluding sprains within 3 months), 2) history of habitual sprains or instability, 3) self-reported ankle instability or function confirmed by a validated questionnaire.
- (2) Interventions: strength training(elastic bands or resistance exercises); balance training(single leg balance or balance board or proprioception); combination of strength and balance training.
- (3) Comparators: control(no exercise) or maintain daily life.
- (4) Outcomes: There was no restriction on the measurement of results, but this meta-analysis focused on the star excursion balance text(SEBT) and self-reported function(FAAM, CAIT, FADI, AOFAS).
- (5) Study design: randomized controlled trials (RCTs).

Articles were excluded if: (1) Trial conducted in animals and acute ankle sprain; (2) Articles that were a case report or detection or survey or not published as peer-reviewed journal articles, such as book chapters and conference abstracts.

### Data extraction

The process of data extraction was conducted independently by two authors (YY.S. and W.L) according to the Cochrane Collaboration Handbook. The data were extracted as follows: basic information (first author, year of publication, characteristics of participants), specific information (measures of intervention, intensity, frequency and duration) and outcome measure appropriate for analysis(SEBT, FAAM, AOFAS, CAIT, FADI and AJFAT scores after intervention). Disagreements were resolved by discussion with a third author (Y.S).

For each included study, the mean and the SD of test and follow-up tests were extracted. If any relevant data was missing, we tried to contact the corresponding author or other authors of that study via email to request it.

### Quality assessment

Literature quality evaluation was conducted using the PEDro scale, which is reliable for evaluating the quality of RCTs and and assessing the risk of bias [14]. The PEDro scale had a total of 10 points (1 point if the corresponding indicators were met, 0 point if the indicators were not met, and the first question were not included in the total score). The results were independently reviewed by two reviewers. Studies with a score above 6 are considered as high quality, and scores of less than 6 for studies would reflect the greater potential for biases to affect results of trial.

### Data synthesis and analysis

The included literature was analyzed using Stata-SE 15 and Revman 5.3 software, including effect sizes, publication bias, subgroup analysis and sensitivity analysis. The results of this study were calculated by comparing the average scores of the experimental group and the control group after the intervention. The standardized mean differences (SMD) and SD with 95% CI were calculated for continuous data. Effect sizes were classified as trivial (< 0.2), small (0.2 ~ 0.5), moderate (0.5 ~ 0.8), or large (> 0.8). A randomized effects model was applied, and the inverse variance method was used. The statistical heterogeneity was evaluated using heterogeneity chi-squared (χ^2^) and *I*^2^ values. The level of heterogeneity was interpreted according to the guidelines from Cochrane,s collaboration: *I*^2^ values of 25, 50, and 75% correspond to low, moderate, and high heterogeneity, respectively [15]. When* I*^2^ ≥ 50%, sensitivity analysis should be performed to test the stability of the results and subgroup analysis should be conducted based on the characteristics of the literature. In addition, We used Egger's test and funnel plot to quantitatively analyze publication bias of the included studies. If a publication bias is observed, the trim and fill methods are used to adjust the estimation points.

## Results

### Literature search and screening

**Fig. 1:**
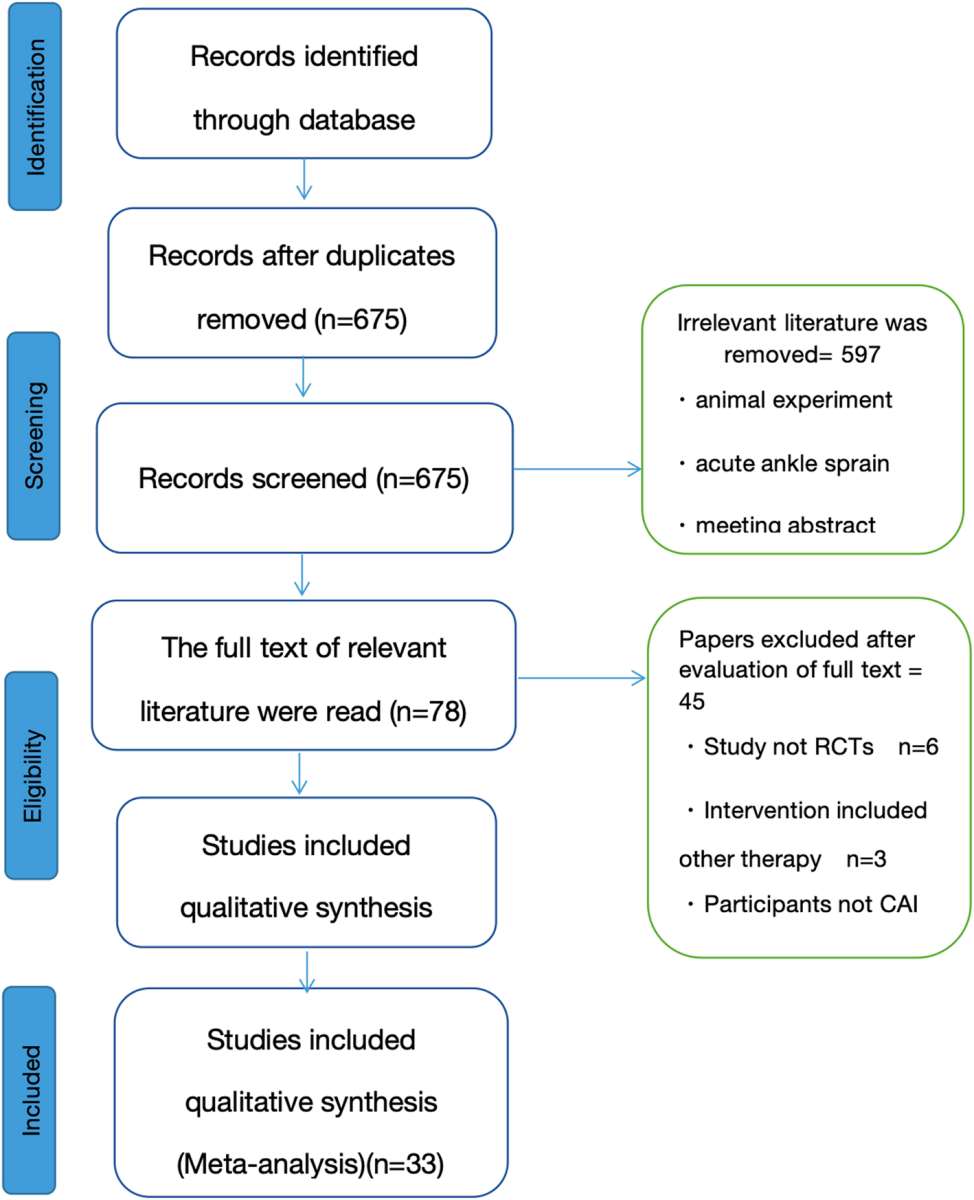
Flowchart of Literature Search and Study Selection

According to PRISMA guidelines, the detailed selection process of these trials were showed in Fig. 1. From the electronic retrieval, a total of 982 relevant studies were obtained from 5 databases, and 597 articles were defined as unqualified after screening by title and abstract. After reading the full text, 33 RCTs were included for Meta analysis, including 11 Chinese literature and 22 English literature [10–12, 16–45] (Table 1).

**Table 1:** Characteristics of Studies Included in Meta-analysis

Author, Year	E/C	Sex	Intervention Program		Duration and Frequency	Main Outcome Measures
		Male/Female	Experimental Group	Control Group		
Jiang, 2020 [16]	30/30	40/20	Combination of strength and balance training	conventional physical therapy	4 WK/6F, 20min	AOFAS
Liang, 2015 [17]	16/16	19/13	Dynamic and static balance training	Maintain routine life	4 WK/5F, 15min	CAIT
Yang, 2014 [18]	32/30	39/23	Combination of strength and balance training	Maintain routine life	4 WK/6F, 24-40min	AOFAS
Liang, 2019 [19]	20/10	0/30	Combination of strength and balance training	Maintain routine life	12 WK/3F, 60min	SEBT
Guo, 2019 [20]	16/8	9/15	Strength training	Maintain routine life	6 WK/3F, 15-20min	SEBT
He, 2014 [21]	13/13	vague	Strength training	Maintain routine life	12 WK/6F, 15-20min	AOFAS
Liu, 2014 [22]	16/16	13/19	Combination of strength and balance training	Family exercise	4 WK/3F, 40min	FADI
Zhang, 2019 [23]	10/10	4/16	Dynamic balance training	2D Dynamic Balance Training	4 WK/4F, 30min	CAIT
Zhu, 2019 [24]	36/12	vague	Combination of strength and balance training	Maintain routine life	10 WK/3F, 60min	AJFAT
Tang, 2022 [42]	27/27	30/30	balance training	BOSU balance training	6 WK/3F, 30min	CAIT
Liu, 2021 [43]	19/18	24/13	Combination of strength and balance training	Strength training	6 WK/3F, 60min	CAIT
Anguish, 2018 [10]	9/9	2/16	Dynamic balance training	Static balance training	4 WK/3F, 30min	FAAM-S,SEBT
Wright, 2017a [11]	20/20	29/11	balance training	Strength training	4 WK/3F, 15min	CAIT,SEBT
Lee, 2019 [12]	15/15	15/15	Dynamic balance training	Static balance training	8 WK/3F, 20-25min	CAIT
Hale, 2007[25]	16/13	10/19	Combination of strength and balance training	Maintain routine life	4 WK/3F, 30min	FADI-S, SEBT
Mckeon, 2008 [26]	16/15	12/19	balance training	Maintain routine life	4 WK/3F, 20min	FADI-S, SEBT
Minoonejad, 2019 [27]	14/14	28/0	Dynamic balance training	Maintain routine life	6 WK/3F, 15-20min	CAIT
Cain, 2017 [28]	11/11	11/11	balance training	Maintain routine life	4 WK/3F, 10-15min	SEBT
Sierra-Guzman, 2018 [29]	33/17	33/17	balance training	Maintain routine life	6 WK/3F, 15min	SEBT
Wright, 2017b [30]	20/10	30/0	Combination of strength and balance training	Maintain routine life	4 WK/3F, 15-20min	CAIT
Cruz-Diaz, 2015 [31]	35/35	35/35	balance training	physical activity	6 WK/3F, 20-25min	CAIT, SEBT
Nam, 2018 [32]	13/15	28/0	Combination of strength and balance training	Strength training	8 WK/3F, 30min	CAIT
Cloak, 2013 [33]	22/11	33/0	balance training	Maintain routine life	6 WK/2F, 15min	SEBT
Kim, 2014 [34]	21/10	7/24	Combination of strength and balance training	Maintain routine life	4 WK/3F, 15min	CAIT
Deussen, 2018 [35]	14/6	14/6	1 texture balance training, 2 smooth balance training	Maintain routine life	6 WK/2F, 20-30min	CAIT
Hall, 2018 [36]	26/13	21/18	1 Balance training, 2 Strength training	Maintain routine life	6 WK/3F, 20min	FAAM-S
Cain, 2020 [37]	32/11	20/23	Strength, balance and combination training	Maintain routine life	4 WK/3F, 15-20min	CAIT,SEBT
Melam, 2018 [38]	15/15	30/0	Strength training	conventional physical therapy	4 WK/4F, 20min	SEBT
Linens, 2016 [39]	17/17	34/0	balance training	Maintain routine life	4 WK/3F, 15min	SEBT
Clark, 2005 [40]	10/9	vague	balance training	Maintain routine life	4 WK/3F, 15min	AJFAT
Collins, 2014 [41]	13/14	10/17	Combination of strength and balance training	conventional physical therapy	4 WK/3F, 15min	FAAM-S, SEBT
Kim, 2021 [44]	25/24	25/24	balance training	Maintain routine life	6 WK/3F, 20min	CAIT,SEBT
Ardakani, 2019 [45]	14/14	28/0	Dynamic balance training	Maintain routine life	6 WK/3F, 30min	CAIT

*E* Experience group, *C* Control group, *WK* weeks, *F* frequency, *CAIT* Cumberland ankle instability tool, *AOFAS* American orthopaedic foot and ankle society, *FADI* foot and ankle disability index, *AJFAT* Functionalassessment tool, *FAAM-S* Functional ankle ability measure-sport, *FADI-S* Foot and ankle disability index-Sport, *SEBT* Star excursion balance text

### Characteristics of included studies

#### Participant characteristics

In the included literature, a total of 1154 subjects were included (Table 1). There were 646 people in the experimental group and 508 people in the control group. Including 633 male subjects and 434 female subjects, However, 3 studies did not state gender [18, 24, 40]. The age distribution of the subjects is between 18–50 years old, and they are all healthy adults.

### Intervention characteristics

The information of the intervention parameters is included in Table 1. Six studies in the experimental group intervention program focused on strength training; twenty studies focused on balance training and Twelve studies focused on combination of strength and balance training. Control group interventions consisted of maintenance of daily routine, no intervention, or strength and balance training. Intervention frequency of 4–12 weeks, 2–6 times a week and 20–60 min each time included in the studies. The main outcome measures of dynamic balance is SEBT and self-reported function score includes AOFAS, CAIT, FADI, AJFAT, FAAM-S and FADI-S.

### Quality assessment of the included studies

Two reviewers independently evaluated the quality of assessment of the included studies. The scores were evaluated according to 9–10 points as high-quality literature, 6–8 points as higher-quality literature, 4–5 as general quality literature [14]. In all 33 literature, 27 higher-quality papers with a score of 6 or above, and 6 general quality papers. Most of the literature are concentrated between 6–8 points, with only one high-quality literature. The conditions for including subjects in the literature are relatively clear, and allocation concealment and blinding methods are rarely used in Chinese literature. Overall, the quality assessment of the included studies are higher in Table 2.

**Table 2:** PEDro Score of The Included Literatures

Author, Year	eligibility criteria	Random Allocation	Concealed Allocation	Groups Similar At Baseline	Participant Blinding	Therapist Blinding	Assessor Blinding	< 15% Dropouts	Intention to Treat Analysis	Between Group Difference Reported	Point Estimate and Variability Reported	Total (0 to 10)
Jiang, 2020 [16]	1	1	0	1	0	0	0	1	1	1	1	6
Liang, 2015 [17]	1	1	0	1	0	0	0	1	1	0	1	5
Yang, 2014 [18]	1	1	0	1	0	0	0	1	1	1	0	5
Liang, 2019 [19]	1	1	0	1	0	0	0	1	1	1	1	6
Guo, 2019 [20]	1	1	0	1	0	0	0	1	1	0	1	5
He, 2014 [21]	1	1	0	1	0	0	0	1	0	1	1	5
Liu, 2014 [22]	1	1	0	1	0	0	0	1	1	1	1	6
Zhang, 2019 [23]	1	1	0	1	0	0	0	1	1	0	1	5
Zhu, 2019 [24]	1	1	0	1	0	0	0	1	0	1	1	5
Tang, 2022 [42]	1	1	0	1	0	0	0	1	1	1	1	6
Liu, 2021 [43]	1	1	0	1	0	0	0	1	1	1	1	6
Anguish, 2018 [10]	1	1	0	1	1	0	0	1	1	1	1	7
Wright, 2017a [11]	1	1	1	1	1	0	0	1	1	1	1	8
Lee, 2019 [12]	1	1	0	1	1	0	0	1	1	1	1	7
Hale, 2007 [25]	1	1	0	1	0	0	0	1	1	1	1	6
Mckeon, 2008 [26]	1	1	1	1	0	0	0	1	1	1	1	7
Minoonejad, 2019 [27]	1	1	1	1	0	0	1	1	1	1	1	8
Cain, 2017 [28]	1	1	0	1	0	0	0	1	1	1	1	6
Sierra-Guzman, 2018 [29]	1	1	1	1	0	0	0	1	1	1	1	7
Wright, 2017b [30]	1	1	0	1	0	0	0	1	1	1	1	6
Cruz-Diaz, 2015 [31]	1	1	1	1	1	0	0	1	1	1	1	8
Nam, 2018 [32]	1	1	0	1	0	0	0	1	1	1	1	6
Cloak, 2013 [33]	1	1	0	1	0	0	0	1	1	1	1	6
Kim, 2014 [34]	1	1	0	1	0	0	0	1	1	1	1	6
Deussen, 2018 [35]	1	1	1	1	0	0	0	1	1	1	1	7
Hall, 2018 [36]	1	1	0	1	0	0	0	1	1	1	1	6
Cain, 2020 [37]	1	1	0	1	1	0	0	1	1	1	1	7
Melam, 2018 [38]	1	1	0	1	0	0	0	1	1	1	1	6
Linens, 2016 [39]	1	1	0	1	0	0	0	1	1	1	1	6
Clark, 2005 [40]	1	1	0	1	0	0	0	1	1	1	1	6
Collins, 2014 [41]	1	1	0	1	0	0	0	1	1	1	1	6
Kim, 2021 [44]	1	1	1	1	1	0	0	1	1	1	1	8
Ardakani, 2019 [45]	1	1	1	1	1	0	1	1	1	1	1	9

## Meta-analysis: comparison of strength, balance and combination training VS. control on self-reported function score

**Fig. 2:**
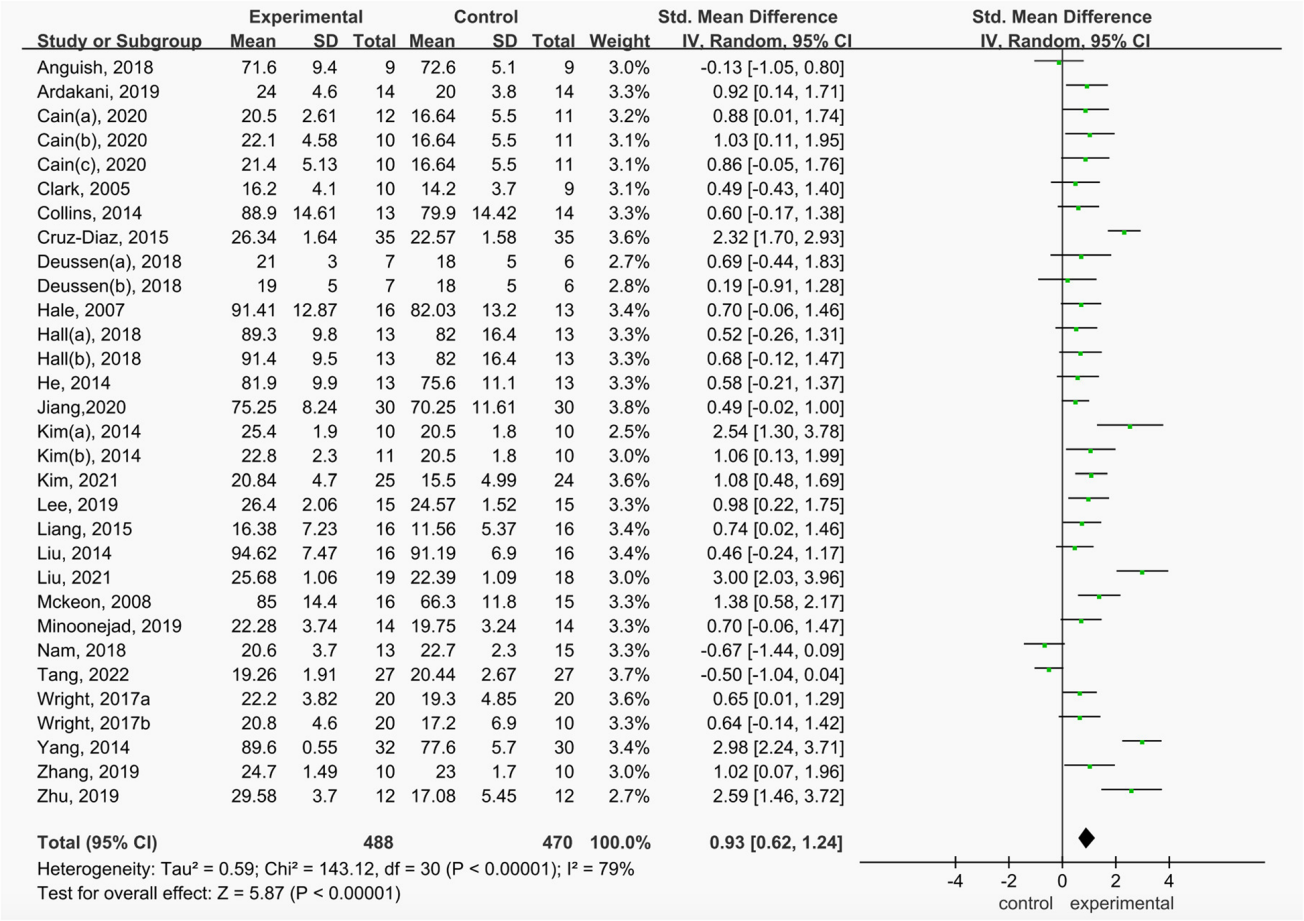
Comparison of the effects of strength, balance and combination training versus control training on self-reported function score

The results the self-reported function score of strength, balance and combination training included 31 studies, including 907 in individuals with CAI. Figure 2 shows significant differences in self-reported function score (SMD = 0.93, 95%CI = 0.62 to 1.24,* p* < 0.05) between the strength, balance and combination training group and control group in this study where this outcome was addressed.

Table 3 shows the subgroup analysis of different covariates on self-reported function score. Strength training (SMD = 0.80, 95%CI = 0.39 to 1.22,* p* < 0.05), balance training (SMD = 0.79, 95%CI = 0.41 to 1.17, *p* < 0.05) and combined training (SMD = 1.28, 95%CI = 0.57 to 1.99, *p* < 0.05) can significantly improve the self-reported function score of CAI patients. Combined training improved to a greater extent compared to strength training and balance training. The subgroups of duration and frequency showed that it was best to improve the function score by 6 weeks of intervention, more than 3 times a week and more than 30 min every exercise.

**Table 3:** Result of Subgroup Analysis on Different Covariates on Functional Score of CAI Patients

Intervention	Subgroup	Studies	SMD (95%CI)	*p*	I^2^
Exercise	Strength training	4	0.80[0.39, 1.22]	*p* < 0.05	0%
	Balance training	16	0.79[0.41, 1.17]	*p* < 0.05	73.8%
	combination training	11	1.28[0.57, 1.99]	*p* < 0.05	88.7%
Duration	4 Weeks	17	0.97[0.61, 1.32]	*p* < 0.05	69.6%
	6 Weeks	10	0.98[0.31, 1.65]	*p* < 0.05	87.1%
	> 6 Weeks	4	0.85[-0.37, 2.07]	*p* > 0.05	88.1%
Frequency	2 Times	2	0.46[-0.33, 1.25]	*p* > 0.05	0%
	3 Times	24	0.94[0.58, 1.30]	*p* < 0.05	79.5%
	> 3 Times	5	1.18[0.25, 2.11]	*p* < 0.05	88.2%
Time of each exercise	t ≤ 20	17	0.82[0.63, 1.00]	*p* < 0.05	0%
	20 < t ≤ 30	11	0.80[0.05, 1.55]	*p* < 0.05	90.2%
	t > 30	3	2.04[0.27, 3.81]	*p* < 0.05	91.0%

*P* Significant difference

I^2^ Heterogeneity

**Fig. 3:**
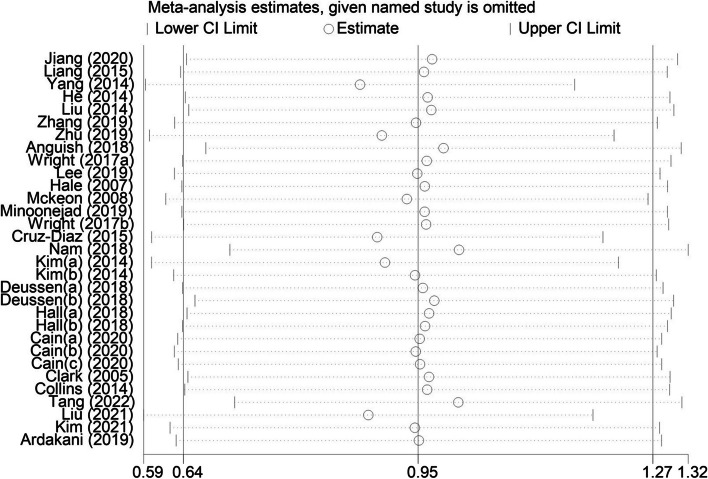
Sensitivity Analysis of exercise intervention on Functional Score of CAI Patients

The heterogeneity I^2^ = 79% in this study, so through sensitivity analysis discussed whether a certain study has a greater impact on the whole. Figure 3 shows that excluding a certain study has little impact on the overall heterogeneity, and the Meta analysis results are stable.

### Meta-analysis: comparison of strength, balance and combination training VS. control on dynamic balance

**Fig. 4:**
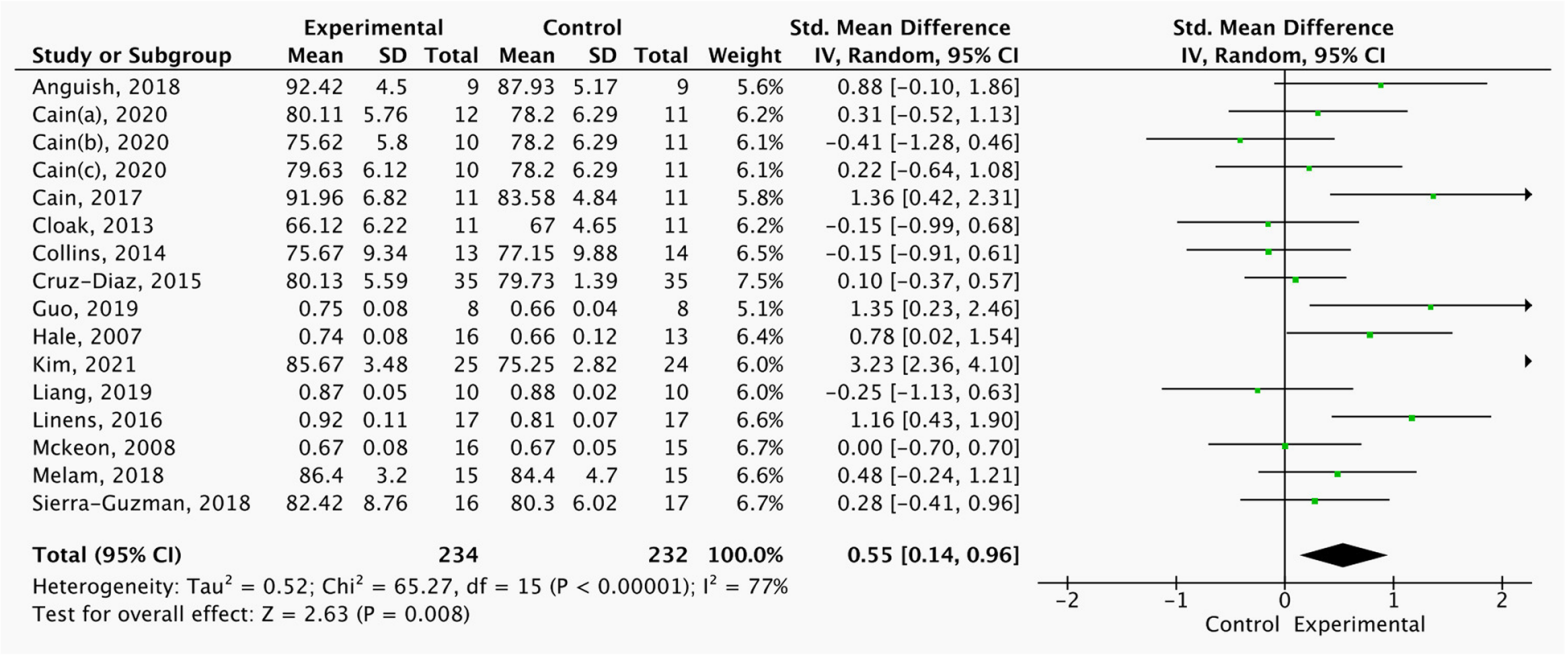
Comparison of the effects of strength, balance and combination training versus control training on SEBT-A

**Fig. 5:**
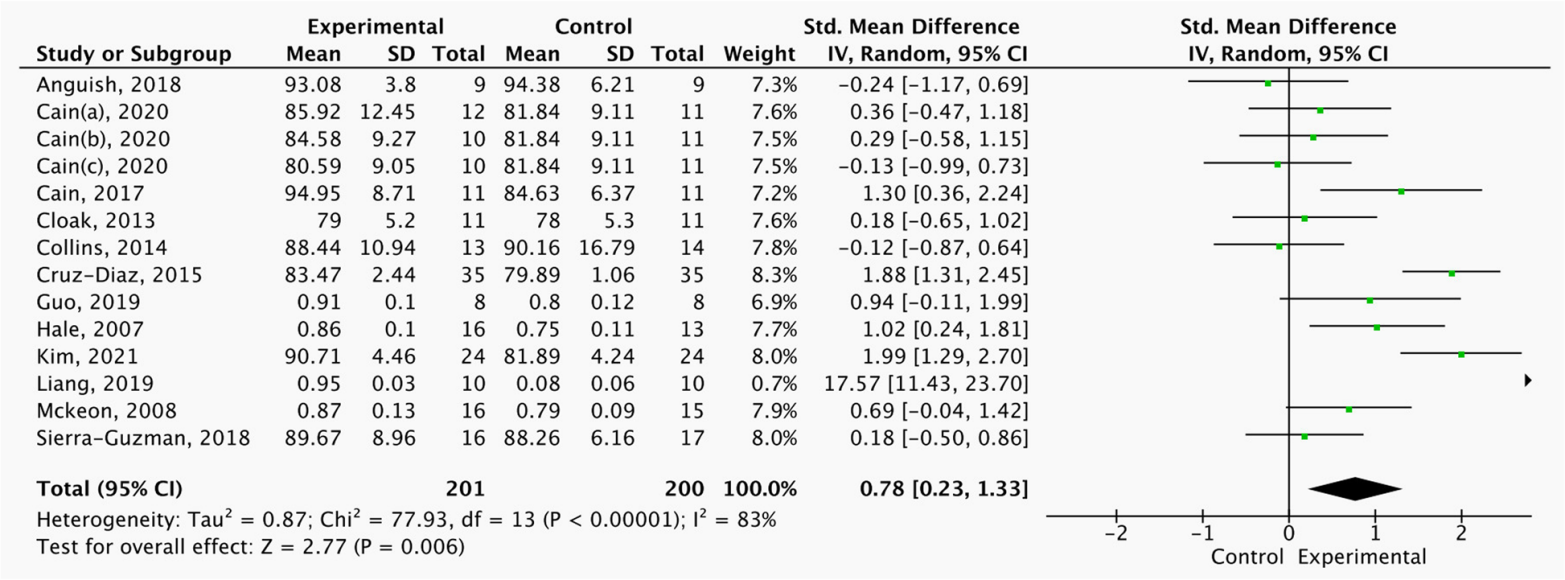
Comparison of the effects of strength, balance and combination training versus control training on SEBT-PL

**Fig. 6:**
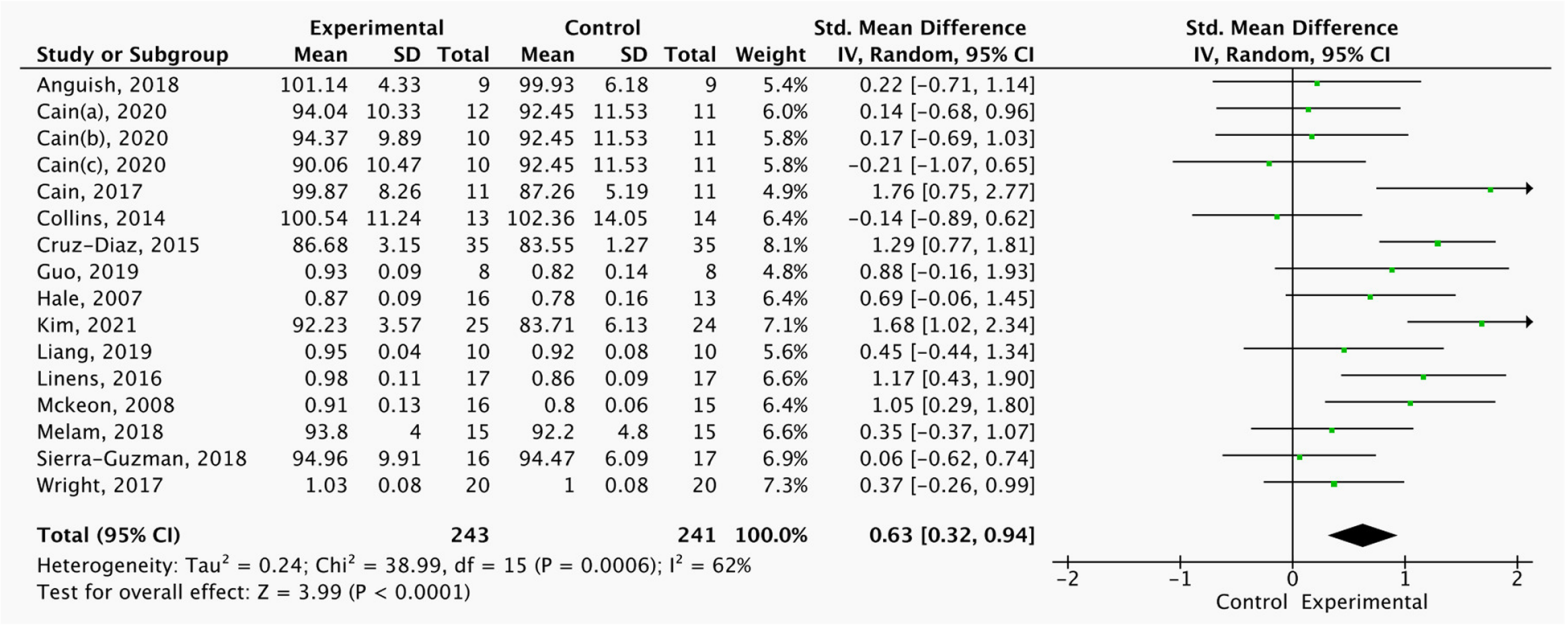
Comparison of the effects of strength, balance and combination training versus control training on SEBT-PM

Figs 4, 5 and 6 shows significant differences in A (SMD = 0.55, 95%CI = 0.14 to 0.96, *p* < 0.05), PL (SMD = 0.78, 95%CI = 0.23 to 1.33,* p* < 0.05), and PM (SMD = 0.63, 95%CI = 0.32 to 0.94,* p* < 0.05) between the strength, balance and combination training group and control group in this study where this outcome was addressed.

Table 4 shows the subgroup analysis of different Covariates on dynamic balance. Strength training can effectively improve SEBT-A, but has no effect on SEBT-PL and SEBT-PM. Balance training can effectively improve the three directions of SEBT-A, SEBT-PL and SEBT-PM, and can achieve moderate effect size. Combination training has no significant improvement effect in three directions. The intervention duration of 6 weeks, three times a week and less than 20 min each exercise were the best combination to improve the dynamic balance of CAI patients.

**Table 4:** Result of Subgroup Analysis on Different Covariates on dynamic balance of CAI Patients

Intervention	Subgroup	A- SMD (95%CI)	PL- SMD (95%CI)	PM- SMD (95%CI)
Exercise	Strength training	0.64(0.07, 1.21) *	0.61(-0.04, 1.26)	0.41(-0.07, 0.89)
	Balance training	0.71(0.03, 1.40) *	0.84(0.22, 1.46) *	0.88(0.45, 1.32) *
	combination training	0.17(-0.31, 0.66)	0.54(-0.24, 1.32)	0.21(-0.25, 0.67)
Duration	4 Weeks	0.96(-0.19, 2.10)	0.42(0.02, 0.82) *	0.51(0.17, 0.84) *
	6 Weeks	0.46(0.10, 0.82) *	1.08(0.25, 1.92) *	1.02(0.30, 1.73) *
Time of each exercise	t ≤ 20	0.64(0.09, 1.19) *	0.59(0.13, 1.05) *	0.61(0.22, 1.00) *
	20 < t ≤ 30	0.50(-0.06, 1.06)	0.95(-0.26, 2.15)	0.84(0.221.46) *
	t > 30	-0.26(-1.14, 0.62)	–––––-	–––––-

*A* Anterior, *PL* Posterolateral, *PM* Posteromedial

^*^ *p* < 0.05

### Publication bias

**Fig. 7:**
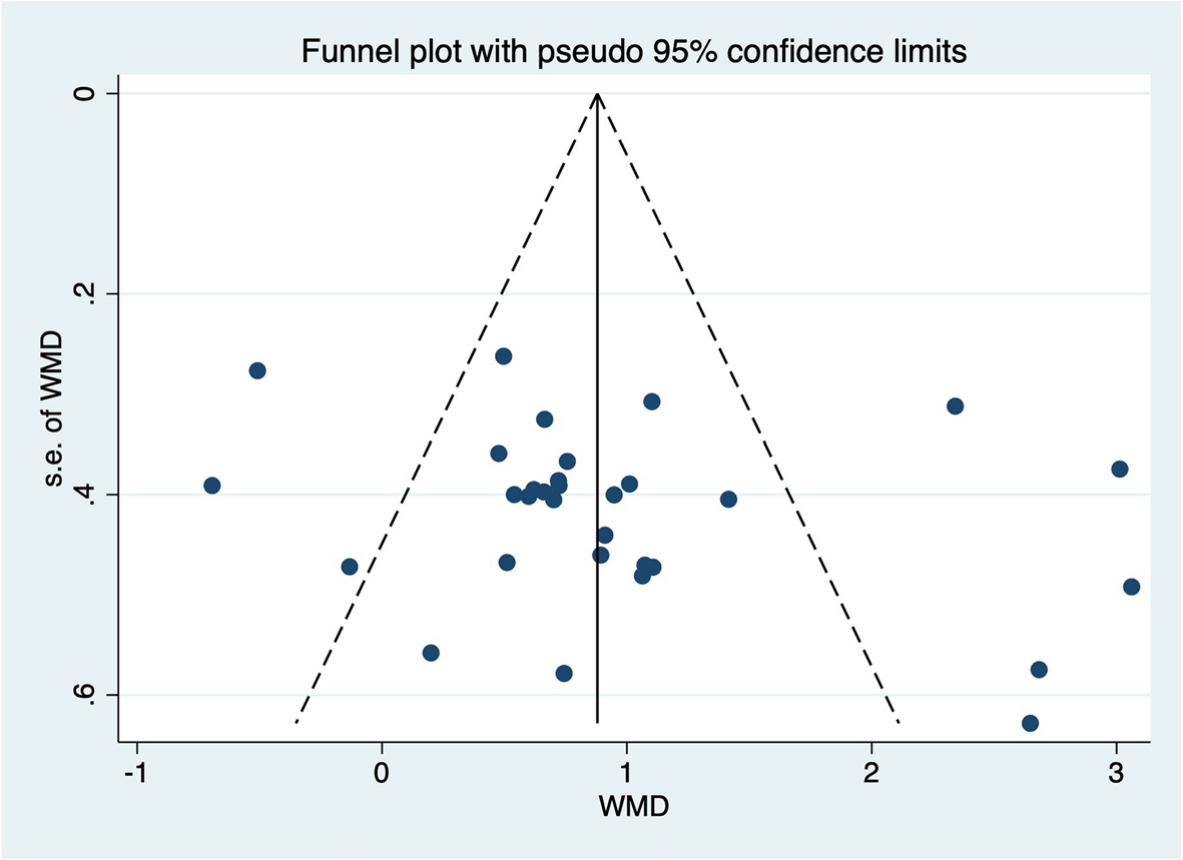
Funnel plot for analyzing the outcome of the self-reported function score, which is symmetrical and indicates no bias of included studies

**Fig. 8:**
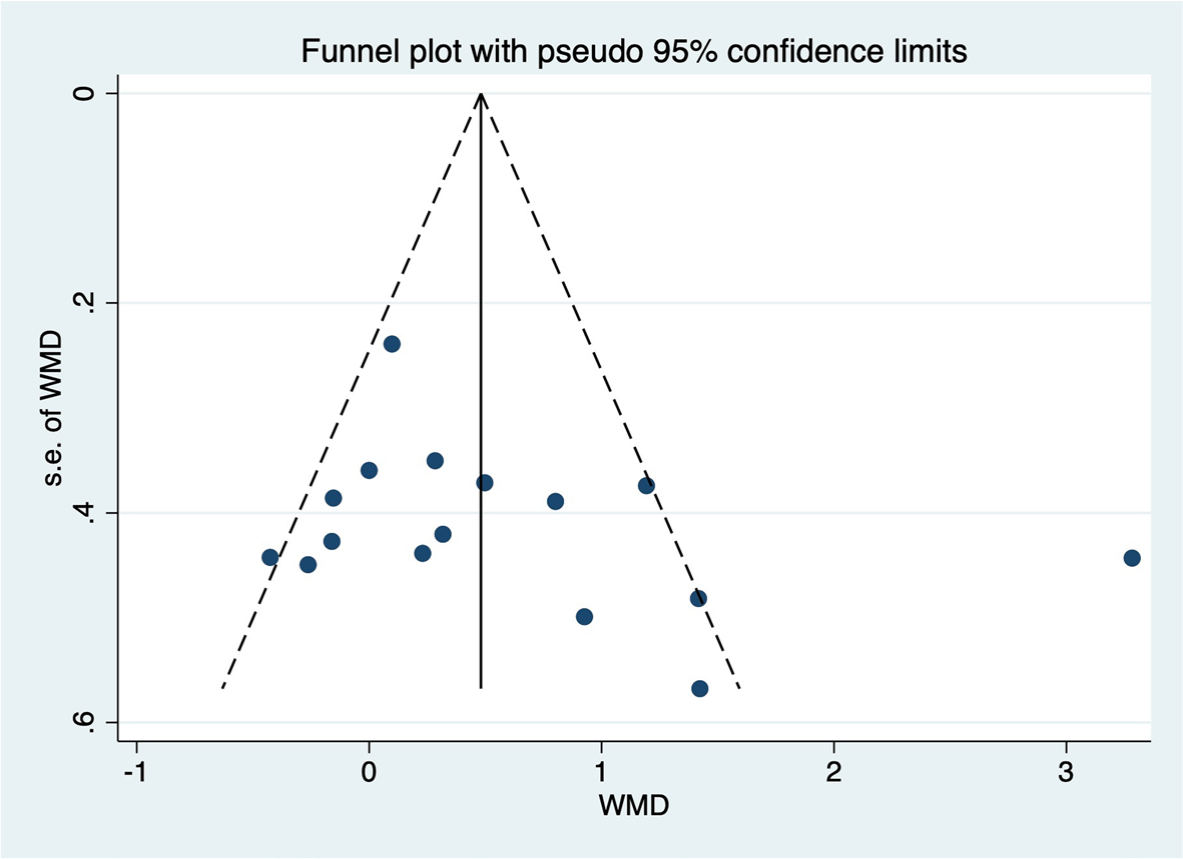
Funnel plot for analyzing the outcome of the SEBT-A, which is symmetrical and indicates no bias of included studies

**Fig. 9:**
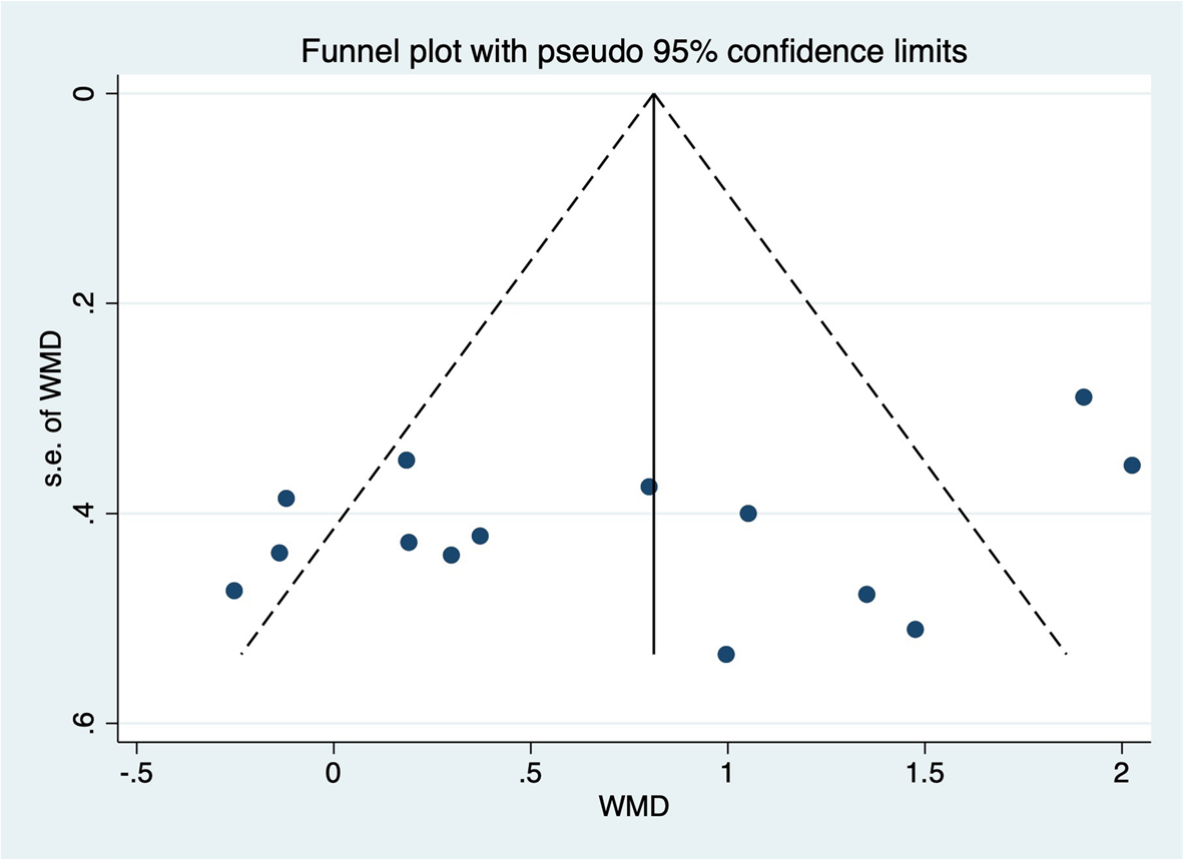
Funnel plot for analyzing the outcome of the SEBT-PL, which is symmetrical and indicates no bias of included studies

**Fig. 10:**
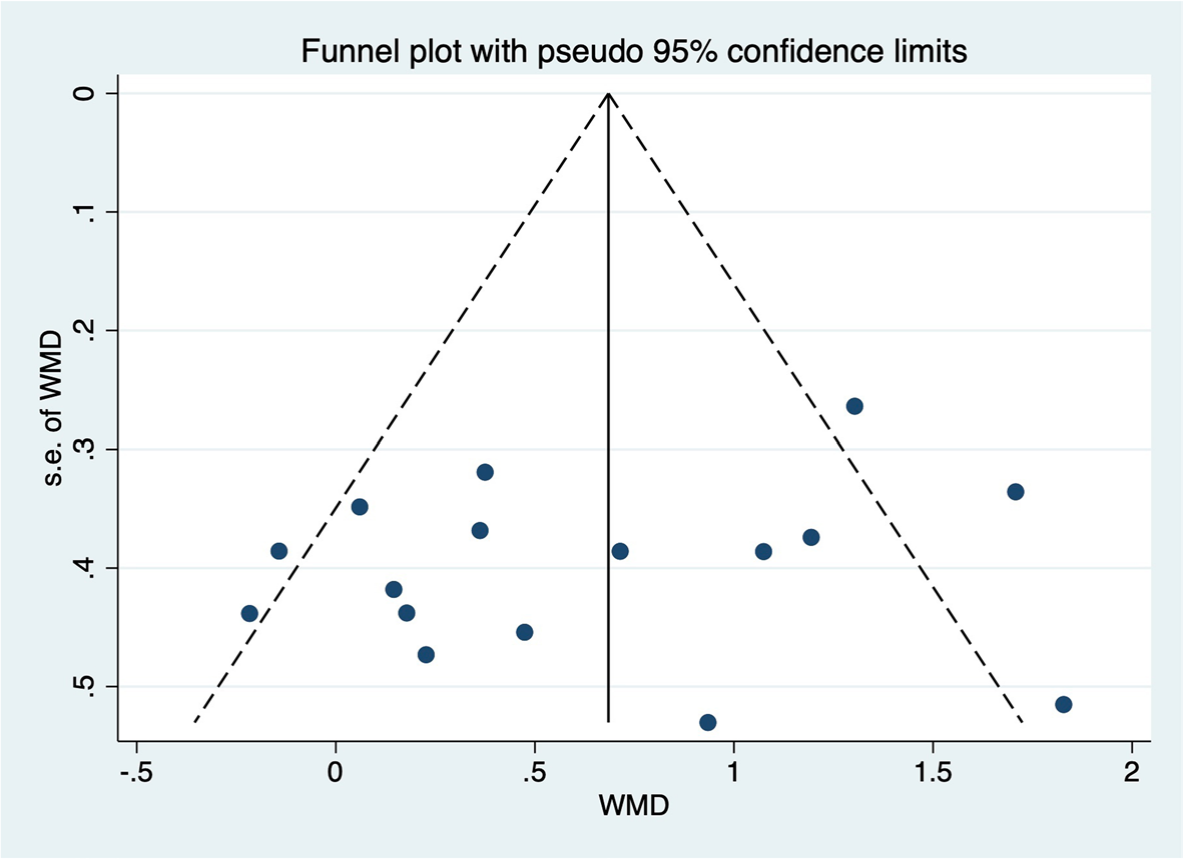
Funnel plot for analyzing the outcome of the SEBT-PM, which is symmetrical and indicates no bias of included studies

The funnel plot and Egger test were used to evaluate the publication bias. No indication of asymmetry or publication bias was found in funnel plot (Figs. 7, 8, 9 and 10). The Egger test in Table 5 showed that there was no publication bias (*p* > 0.05) in the self-report function (*p* = 0.147), SEBT-A (*p* = 0.204), SEBT-PL (*p* = 0.158), and SEBT-PM (*p* = 0.331).

**Table 5:** Meta Analysis of Egger Test Result

outcome measures	Std_Eff	Coef	Std. Err	*t*	*p*	[95%Conf. Interval]
self-reported function	Slope	-0.2385	0.7664	-0.31		-1.8062, 1.3290
	Bias	2.8891	1.9390	1.49	0.147	-1.0765, 6.8548
SEBT-A	Slope	-0.8221	0.9987	-0.82		-2.9641, 1.3200
	Bias	3.3947	2.5468	1.33	0.204	-2.0675, 8.8570
SEBT-PL	Slope	2.7029	1.2736	2.12		-0.0719, 5.4778
	Bias	-4.7478	3.1553	-1.50	0.158	-11.6227, 2.1270
SEBT-PM	Slope	1.556	0.8804	1.77		-0.3322, 3.4442
	bias	-2.3088	2.2950	-1.01	0.331	-7.2312, 2.6136

## Discussion

### Patient reported outcomes

Self-reported function can diagnose the rehabilitation effect of CAI patients, generally including pains, swellings, losses of control and other symptoms, as well as the performance of daily life related to running, jumping, landing and squatting. The clinical evaluation of CAI rehabilitation effect by patient reported outcomes has the advantages of saving time and being fast and effective. The scale has been proved to have high test efficiency [4, 46]. A total of 31 literatures including 907 CAI patients were included in the patient reported outcomes. The strength training, balance trainging, and combined strength and balance training can significantly improve the chronic ankle instability self-reported function compared to the control group. Combined strength and balance training improved to a greater extent compared to strength training and balance training. The best exercise intervention for improving self-reported function is to exercise for 6 weeks, more than 3 times a week and more than 30 min every exercise. Chronic ankle instability often presents with deficits in neuromuscular control, proprioception, and strength disorders [47]. These symptoms can affect people's daily quality of life, limit physical exercise, and cause pain [11]. Because of the incidence rate and commonness of chronic ankle instability, people try to improve it by providing effective technology. Physical exercise is an effective physical method for treating chronic ankle instability, and balance [45, 48] and strength [36, 49] are common exercise interventions. So this study can provide guidance and recommendations for clinical treatment and design.

Compared to previous literature, previous studies have investigated the effects of balance training and strength training on CAI, but have not compared strength training, balance training, and combined strength and balance training interventions. Luan et al. [8] concluded that strength training did not improve FAAM compared to the control group and did not include other self-reported function indicators, so only two literature were included. Koshino et al. [50] compared the effects of several exercise interventions on dynamic balance, but did not include self-reported function. Only one literature compared the effects of strength training and balance training on self-reported function and dynamic balance in their research, and concluded that balance training significantly improved the self-reported function and dynamic balance. Compared to strength training, balance training can better improve self-reported function, but has no advantage in improving dynamic balance [51].

The main causes of CAI symptoms may include dynamic balance ability, proprioception, fibular reaction time and loss of valgus force [7]. The loss of dynamic balance and proprioception will cause CAI patients to have symptoms such as blocked nerve information input, weakened muscle control, and muscle lack strength [46]. Posture control is affected by the input of information such as vision, vestibular sense, position sense and proprioception. Therefore, posture defects are likely to be affected by the impairment of neuromuscular control and proprioception. It is beneficial to improve the obstacle of posture control, neuromuscular control and proprioception through dynamic and static balance training [52, 53].

The decline of valgus strength of ankle joint is more likely to cause muscle weakness, ligament relaxation and repeated ankle sprains. The valgus muscle strength of the ankle joint can provide protection for the lateral ankle ligament to resist the varus force. There is a high correlation between valgus muscle loss and chronic ankle instability [54]. The risk of the ankle sprain can be reduced by strengthening ankle muscle strength after ankle sprain. In clinical practice, it is generally recommended using elastic band and resistance exercise to improve valgus and dorsiflexion strength [55]. We can think that the combined strength and balance training will be more effective and targeted than the single form of strength and balance training in posture control, dynamic balance improvement and related CAI symptom reduction. This conclusion is also supported by the systematic meta-analysis results of Mollà-Casanova et al. [51]. Balance training may help to improve ankle function and reduce incidence rate, and the effect is similar to that of strength training. The combination of two training methods yields better results [19]. Therefore, it is recommended that rehabilitation practitioners and clinicians use the combination of strength and balance training to rehabilitate the ankle joint, which is the best way to improve the patient's symptoms.

### Dynamic balance

SEBT was significantly correlated with postural control and dynamic balance ability of the lower limbs. The ability of dynamic balance was evaluated efficiently by SEBT score [56]. This study found that strength training had a significant improvement on SEBT-A, but not on SEBT-PL and SEBT-PM. Balance training had a significant improvement in all three directions and achieved moderate effect size. The combination of strength and balance training had no significant improvement on the three directions. Dynamic balance is the ability of the center of gravity of the body to maintain postural stability and orientation in the plane of support while the body is in motion. In the process of human movement, the support plane of the body is constantly changing, and the body posture is constantly adjusted.

Research by Linens [39] and Anguish [10] suggested that balance training had the best effect in improving SEBT scores. This study also found that balance training significantly improved dynamic balance ability, while strength training may have a low effect on dynamic balance improvement. Neuromuscular control and proprioceptive reconstruction recovery are prerequisites for improving postural stability [5]. Balance training requires better control of body stability in both dynamic and static exercise. This is helpful for neural control and proprioception. Dynamic balance is more dependent on the control of body posture, and has great relevance for trunk posture, lower limb muscle strength [52]. Strength training generally targets the muscles around the ankle joint to improve results. Strength training needs to be carefully chosen to target improvements in neural control and proprioception.

The actions and control in human movement depend on the sensorimotor system, which integrates the nervous system with the sensory system, forming a complex process of integration [6]. When the ankle sustains a sports injury, damage occurs to the muscles, tendons, and proprioceptors of the ankle joint, impeding the incoming information. The diminished neuro-muscular control, originally responsible for executing the movement, leads to weakened capabilities, making the ankle joint more susceptible to recurrent sprains and secondary injuries during physical activity [17]. When the ankle is sprained, the recovery of neuro-muscular control and proprioceptive sensation is the physiological foundation for effective rehabilitation [57]. The balance training program, including dynamic and static exercises, can enhance postural control in unstable conditions, strengthen neuro-muscular control, and reduce the risk of ankle sprains [25, 58].

## Conclusion

Available evidence showed that, the combination of strength and balance training achieves greater benefits for patient reported outcomes and intervention for 6 weeks, more than 3 times a week and more than 30 min each time were the best rehabilitation programs to improve CAI patientreported outcomes. balance training could bring greater benefits to dynamic balance. Strength training should be used cautiously in clinic to improve the dynamic balance in individuals with CAI.

### Limitations of the study

First of all, some studies included in this study did not show the implementation of allocation concealment and blinding, which may reduce the reliability of the study results or cause subjective bias. Second, the balance training intervention program included single-leg balance training, unstable balance training and other balance interventions, etc. This study did not conduct a more detailed subgroup analysis to determine the effect of different forms of balance intervention. The last, the load and patient reported outcomes measurements of the interventions included in the studies were different, which may lead to bias in the results.

## Data Availability

The research provides the best intervention plan for the rehabilitation of chronic ankle instability through a combination of strength and balance training; 2 Balance training could bring greater benefits to dynamic balance, and strength training should be used cautiously in clinic to improve the dynamic balance in individuals with CAI. The data supporting the results of this study can be obtained from the 33 RCTs literature included.
